# RETRACTION: Fracture‐Related Wound Infections Following Surgical Treatment of Fractures in HIV‐Positive and HIV‐Negative Patients: A Meta‐Analysis

**DOI:** 10.1111/iwj.70383

**Published:** 2025-03-21

## Abstract

**RETRACTION:** ChenH. , ZhuY. , and ChenQ. , “Fracture‐Related Wound Infections Following Surgical Treatment of Fractures in HIV‐Positive and HIV‐Negative Patients: A Meta‐Analysis,” International Wound Journal 21, no. 1 (2024): e14336, 10.1111/iwj.14336.37705307 PMC10784629

The above article, published online on 13 September 2023, in Wiley Online Library (http://onlinelibrary.wiley.com/), has been retracted by agreement between the journal Editor in Chief, Professor Keith Harding; and John Wiley & Sons Ltd. Following an investigation by the publisher, all parties have concluded that this article was accepted solely on the basis of a compromised peer review process. The editors have therefore decided to retract the article. The authors did not respond to our notice regarding the retraction.

**RETRACTION:** H. Chen , Y. Zhu , and Q. Chen , “Fracture‐Related Wound Infections Following Surgical Treatment of Fractures in HIV‐Positive and HIV‐Negative Patients: A Meta‐Analysis,” International Wound Journal 21, no. 1 (2024): e14336, 10.1111/iwj.14336.37705307 PMC10784629

The above article, published online on 13 September 2023, in Wiley Online Library (http://onlinelibrary.wiley.com/), has been retracted by agreement between the journal Editor in Chief, Professor Keith Harding; and John Wiley & Sons Ltd. Following an investigation by the publisher, all parties have concluded that this article was accepted solely on the basis of a compromised peer review process. The editors have therefore decided to retract the article. The authors did not respond to our notice regarding the retraction.

